# Flow in fetoplacental-like microvessels in vitro enhances perfusion, barrier function, and matrix stability

**DOI:** 10.1126/sciadv.adj8540

**Published:** 2023-12-22

**Authors:** Marta Cherubini, Scott Erickson, Prasanna Padmanaban, Per Haberkant, Frank Stein, Violeta Beltran-Sastre, Kristina Haase

**Affiliations:** ^1^ European Molecular Biology Laboratory (EMBL), Barcelona, Spain.; ^2^ Proteomics Core Facility, EMBL Heidelberg, Heidelberg, Germany.

## Abstract

Proper placental vascularization is vital for pregnancy outcomes, but assessing it with animal models and human explants has limitations. We introduce a 3D in vitro model of human placenta terminal villi including fetal mesenchyme and vascular endothelium. By coculturing HUVEC, placental fibroblasts, and pericytes in a macrofluidic chip with a flow reservoir, we generate fully perfusable fetal microvessels. Pressure-driven flow facilitates microvessel growth and remodeling, resulting in early formation of interconnected and lasting placental-like vascular networks. Computational fluid dynamics simulations predict shear forces, which increase microtissue stiffness, decrease diffusivity, and enhance barrier function as shear stress rises. Mass spectrometry analysis reveals enhanced protein expression with flow, including matrix stability regulators, proteins associated with actin dynamics, and cytoskeleton organization. Our model provides a powerful tool for deducing complex in vivo parameters, such as shear stress on developing vascularized placental tissue, and holds promise for unraveling gestational disorders related to the vasculature.

## Teaser

Advanced 3D fetoplacental-like vascular model reveals improved barrier function and matrix remodeling in response to flow.

## INTRODUCTION

Human placentae are highly vascularized organs that undergo constant vascular growth and remodeling to ensure effective exchange between maternal blood and the fetal circulatory system (*1*). The majority of the essential exchange of solutes (oxygen, nutrients, hormones, and antibodies) takes place across a thin layer of epithelial cells of the terminal villous trees, which contains a network of fetoplacental capillaries. This dense capillary network develops from the elongation and ramification of preexisting blood vessels formed by vasculogenesis (new vessel formation) by approximately 6 weeks of gestation. From 25 weeks after conception until term, villous vascular growth switches from branching angiogenesis to nonbranching angiogenesis generating coiled capillary structures that reside within the extremities of the fetoplacental vascular trees (*2*).

Formation of a proper vascular tree in the early placenta is crucial to ensure optimal blood volume loading, preventing placental insufficiency and adverse fetal outcomes. Structural villous microvascular network abnormalities are common features of gestational disorders associated with perinatal morbidity and mortality such as in preeclampsia and fetal growth restriction (*3*, *4*). To determine the etiology of these alterations, it is crucial to understand the mechanisms underlying fetoplacental vasculogenesis and regulation.

Studying fetoplacental vascular development is challenging, mainly because of ethical considerations and inaccessibility of the tissue, particularly in early pregnancy. Current knowledge on villous vascular network development and function comes from observations of human placenta explants obtained at different stages of gestation (*5*, *6*), ultrasound examinations (*7*), and in silico modeling (*8*, *9*). However, these methods cannot provide a direct measurement of the microenvironmental impact on placental vasculogenesis and angiogenesis, such as mechanical shear stress and associated mechanotransduction. Developing vessels, in vivo, are exposed to constant mechanical stimuli including fluid shear stress induced by interstitial flow (IF) and intraluminal blood flow (*10*). Several studies have documented the effects of flow-induced shear stress on vascular morphogenesis, demonstrating flow-induced regulation of angiogenic sprouting and lumen formation by endothelial cell migration, alignment, and apical deformation (*11*–*14*). Hemodynamic forces trigger vessel remodeling through cellular rearrangement and regulate vascular permeability via modulation of endothelial adherens and tight junctions (*15*–*18*). The role of hemodynamics during pregnancy remains poorly understood because in vivo experimentation has mainly focused on umbilical circulation of the major artery and vein (*19*), lacking proper characterization of the placental microhemodynamics occurring at the fetoplacental interface. Although the placenta at term constitutes a valid tool for ex vivo perfusion studies (*20*), maintaining their viability is complex, and understanding of early placental vascular circulation is limited, requiring the employment of biomimetic models.

Tissue-specific three-dimensional (3D) microvascular networks have recently contributed to our understanding of flow-induced vascular growth and remodeling by overcoming the limitations of simpler monolayer systems (*14*, *16*, *21*, *22*). For instance, IF has been shown to enhance vessel formation, function, and longevity in brain-specific microvasculature composed of endothelial cells, pericytes, and astrocytes (*23*). Despite the development of novel in vitro placental models [reviewed in (*24*)], at present, no models have been used to investigate the effect of flow-associated placental vascular development. Previously, our 3D model of terminal villi microvasculature was established using a triculture of stromal and endothelial cells (*25*). This model demonstrated that placental pericytes contribute to growth restriction, which was shown to be largely dependent on vascular endothelial growth factor (VEGF) and angiopoietin/Tie2 signaling (*25*). Fibroblasts, on the other hand, contributed to increased vasculogenesis; however, a limitation of our former model was the use of non-specific fibroblasts. To build on our former study, placental fibroblasts are now included in a completely placental-derived human 3D vascular model. Our results indicate that growth and remodeling of fetal-derived microvessels, as well as endothelial barrier function (permeability to solutes) and extravascular matrix (EVM) properties (diffusivity, stiffness, and matrix proteins), are strongly regulated by increasing shear stress. Computational fluid dynamics (CFD) predicts luminal and interstitial shear stresses, which are highly heterogeneous within a single microtissue. Flow-conditioned fetal microvessels exhibit proinflammatory profiles and early connection of vessel networks that remain perfusable for weeks in culture.

Overall, fluid dynamics strongly regulate the development of fetoplacental-like vessels, suggesting that poor or restrictive flow conditions can negatively affect vasculogenesis. Our findings serve as a basis for further research into the mechanisms of placental vascular defects in pregnancy-related disorders related to flow insufficiency.

## RESULTS

### Interstitial flow enables placental-like microvascular network formation and function

**Fig. 1.**
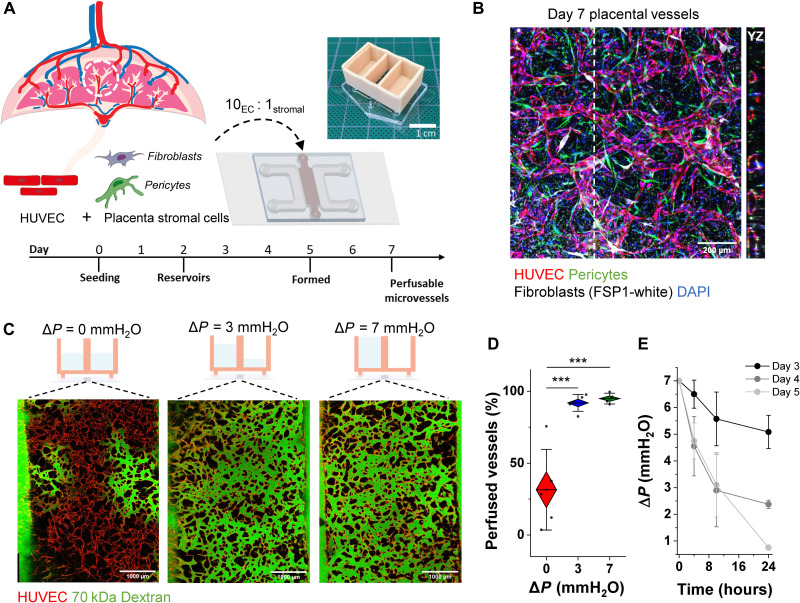
Interstitial flow promotes network perfusion in placental microvessels. (**A**) Schematic demonstrating the culture timeline and protocol. Top right inset: A picture of the assembled device and reservoir. (**B**) Immunofluorescence maximum-projection image of triculture placental vessels with orthogonal view showing 3D open vascular lumen. Dashed line indicates the plane of intersection. Scale bar, 200 μm. (**C**) Interstitial flow conditions (depicted graphically above) promote complete vascular bed perfusion. Perfusion is shown by FITC-dextran introduced into the red fluorescent protein (RFP)–labeled vessels via the media channels (partially shown) at day 7. Scale bar, 1000 μm. (**D**) Percentage of perfused vessels is quantified across static and IF conditioned vessels at day 7. Shown are box plots with outer edge as SE and bars with SD. Significance is measured by one-way analysis of variance (ANOVA) and indicated as ***P < 0.001 for Tukey means comparison test. (**E**) Pressure drop (from Δ*P*_initial_ = 7 mmH_2_O) measured over time across different days in culture. Shown is the mean and SD of three separate experiments with six or more devices.

We previously presented a 3D in vitro model of placental terminal villi microvessels capable of recapitulating several aspects of placental vasculopathies (*25*). Now, to better approximate the physiological cellular composition of placental fetal tissue, we sourced primary placental fibroblasts that were integrated together with endothelial cells [human umbilical vein endothelial cells (HUVECs)] and placental pericytes to generate microvascular tissues on-chip. Cells were cultured with an endothelial-to-stromal cell ratio of 10:1 in fibrin gel at 3.5 mg/ml, as opposed to the 5:1 ratio and 3 mg/ml used previously (*25*), within a single-gel channel device (Fig. 1A). These differences in culture are in part attributed to the different secretome of stromal cells isolated from placental and lung tissues, which show dissimilarities in angiogenic profile (fig. S1). In particular, inflammatory molecules including platelet-derived growth factor BB (PDGF-BB) and monocyte chemoattractant protein-1 (MCP1) are increased for placental fibroblasts in contrast to lung fibroblasts. The formation of connected microvascular networks (with this new triculture) occurs over approximately 1 week (Fig. 1B); however, networks are fully perfusable only when an IF is applied across the hydrogel through the addition of a 3D printed media reservoir (shown in Fig. 1A top right and fig. S2). Placental microvessels cultured under static conditions self-assemble by day 5; however, they were not perfusable, showing loss of connectivity and vessel pruning by day 7 (fig. S3).

Pressures gradients (Δ*P*) representative of static (0 mmH_2_O), low (3 mmH_2_O or ~30 Pa), and high flow (7 mmH_2_O or ~70 Pa) culture conditions were applied to microvessels (Fig. 1C), with mean interstitial fluid flow velocities measured as 0.13 ± 0.06, 0.30 ± 0.12, and 1.23 ± 0.32 μm/s, respectively, at day 2 (fig. S4). On day 7, perfusion with fluorescein isothiocyanate (FITC)–dextran shows complete vessel connectivity in devices cultured under flow, whereas partial perfusion was observed in its absence (Fig. 1C), quantified as perfused vessel area (Fig. 1D).

Vessels form over several days in culture to produce open perfusable lumen (of ~40 μm in diameter at day 7); hence, there is a shift from interstitial to luminal flow. This transition was measured by the fluid volumetric drop (every ≍4 hours). The pressure gradient decays slowly at earlier time points but demonstrates a quick drop over 24 hours by day 5. This suggests the presence of open lumen and a switch from interstitial to primarily intraluminal flow (Fig. 1E).

Early placental development occurs under low oxygen conditions (~2.5% O_2_) and gradually increases (~8% O_2_) from the 14th week of gestation onward (*26*). To confirm that O_2_ concentration would not significantly affect our results, we cultured our microvessels in a low-oxygen environment (8% O_2_). Fetoplacental-like microvessels displayed similar morphology and no change in endothelial permeability compared to those cultured at 21% O_2_ conditions (fig. S5), suggesting that our cells adapted to oxygen changes. Thus, for practical reasons, all further experiments were performed under 21% O_2_ conditions.

### Interstitial flow promotes early placental vasculogenesis

**Fig. 2.**
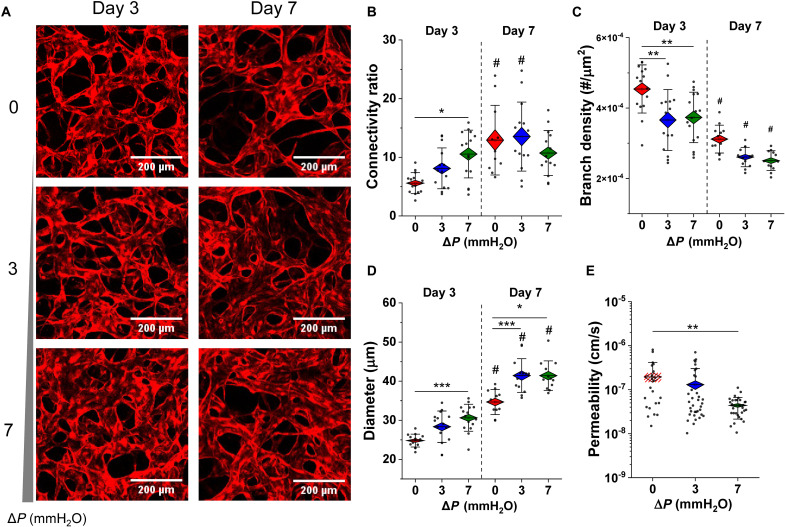
Interstitial flow promotes vessel connectivity and barrier function. (**A**) Confocal images of microvessels (HUVEC with cytoplasmic RFP) at days 3 and 7 of culture under static and flow conditions. Morphology is measured by segmentation of images of RFP-cytoplasmic labeled HUVEC. Morphologic parameters were measured across days 3 and 7 for (B to D). (**B**) Connectivity is quantified as a ratio of vessel junctions to end points. (**C**) Branch density is the number of branches per area. (**D**) Effective diameter is measured as vascular area coverage over length. (**E**) Permeability to 70-kDa dextran is shown for static and flow conditions at day 7. Hashed lines for the static condition indicate that very few vessels were perfused and thus measured. Significance is measured by one-way ANOVA and indicated by *P < 0.05, **P < 0.01, and ***P < 0.001 for Tukey means comparison test and # represents significance across days.

Placental microvessels become increasingly perfusable when subjected to flow; thus, morphologic and barrier properties were assessed. Development of the vessels was monitored at specific time points by confocal microscopy under static, low- and high-flow conditions (Fig. 2A). In early culture (day 3), IF-conditioned vessels showed increased network connectivity (Fig. 2B), reduced branch density (Fig. 2C), and larger vessel diameters (Fig. 2D), statistically significant for high-flow conditions. At day 7, connectivity remained unchanged between flow and static conditions, although values of connectivity were increased over time for static cultured vessels. At this later time point, branch density was reduced in all cases compared to day 3 under the same conditions (Fig. 2C). Although diameter continues to increase over time (from days 3 to 7) across both conditions, flow-conditioned microvessels are significantly larger in diameter compared to static cultured vessels, for both low and high flow (Fig. 2D).

Next, endothelial barrier function was examined by perfusion on day 7 with 70-kDa FITC-dextran. Vessels cultured under high flow conditions, in comparison to static culture, resulted in significantly decreased permeability values (increased barrier function; Fig. 2E), indicating that an intermittently applied pressure gradient is sufficient to enhance vascular barrier stability.

### Interstitial flow enhances inflammatory signaling in developing placental-like microvessels

**Fig. 3.**
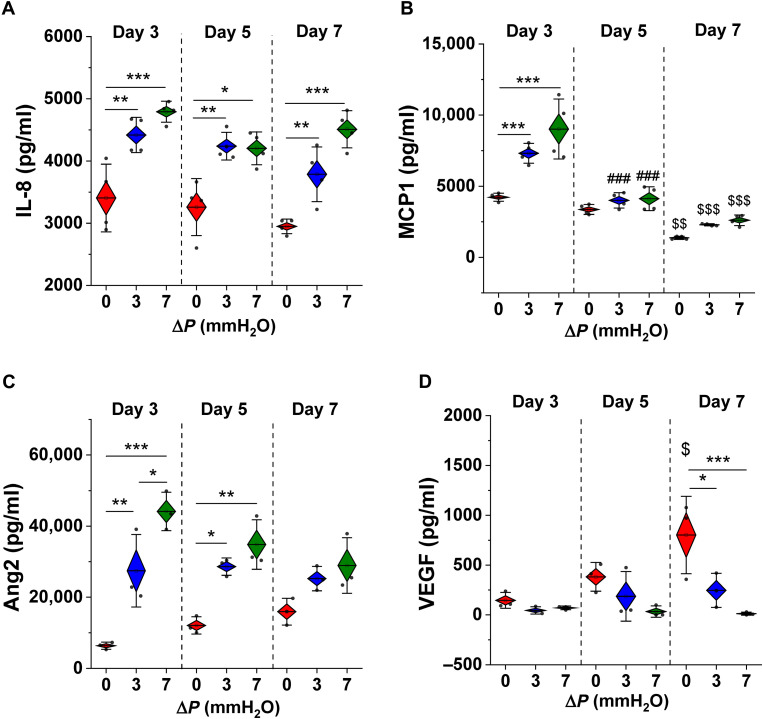
Interstitial flow affects inflammatory and angiogenic signaling at early time points in vessel development. Concentrations of inflammatory molecules (**A**) IL-8 and (**B**) MCP1 were measured by ELISA across various time points from 3D microvessels. Angiogenic signaling molecules (**C**) Ang2 and (**D**) VEGF were significantly different from statically cultured vessels at early and later time points, respectively. Significance is measured by one-way ANOVA and indicated by *P < 0.05, **P < 0.01, and ***P < 0.001 for Tukey means comparison test; # represents significance across day 5 and day 3 and $ across day 7 and day 3.

Vessel formation and remodeling is intimately linked to inflammation and growth factor signaling, yet little is known about signaling in human fetoplacental vasculogenesis. Thus, cytokine expression was examined from static and flow-conditioned placental-like microvessels. Inflammatory cytokines, including interleukin-8 (IL-8) and MCP1, and proangiogenic factors, including VEGF and angiopoietin 2 (Ang2), were quantified by enzyme-linked immunosorbent assay (ELISA; Fig. 3). Generally, flow conditioning (low and high flow) resulted in increased inflammatory signaling across all time points, particularly for IL-8 (Fig. 3A), whereas MCP1 levels (Fig. 3B) dropped over time (although this was shown for both flow and static conditions). Ang2 is up-regulated in the case of flow-conditioned vessels (Fig. 3C), yet VEGF levels are significantly increased in statically cultured vessels over time (Fig. 3D).

### Flow sustains placental microvascular longevity

**Fig. 4.**
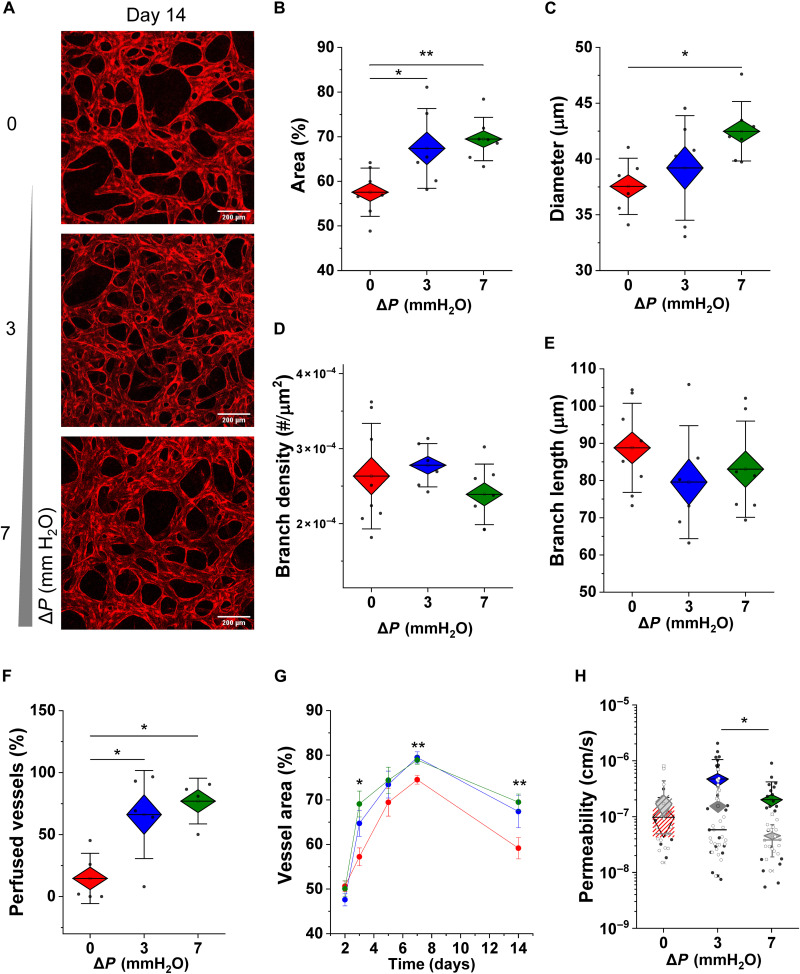
Flow promotes longevity of placental microvessels. (**A**) Confocal images demonstrate differences in vascular density between static and flow-conditioned vessels at day 14 in culture. Both vessel (**B**) area coverage and (**C**) effective diameter are significantly higher for flow-conditioned microvessels. (**D**) Branch density and (**E**) length were not significantly affected by flow. Flow sustains vessel perfusion capacity after 14 days in culture, as seen by (**F**) the high percentage of perfused vessels, and (**G**) higher area coverage overtime than for static conditions. (**H**) Vascular permeability is measured for fully perfused flow-conditioned vessels and the few perfusable vessels that remain in the static condition. Gray diamond boxes display permeability measurements at day 7. Scale bars, 200 μm. Significance is measured by one-way ANOVA and indicated by *P < 0.05 and **P < 0.01 for Tukey means comparison test.

We examined whether flow conditioning could maintain microvascular networks and their functionality in cultures of placental-like microvessels for 1 week following initial perfusion (at day 7). Confocal images acquired at 14 days after seeding demonstrated distinct differences in vessel coverage between static and flow conditions (Fig. 4A). Microvessels maintained under static culture exhibited significantly reduced area coverage (Fig. 4B) and correspondingly narrow effective diameters (Fig. 4C). Branch density and length remained unchanged between static and flow conditions (Fig. 4, D and E).

Perfusion with 70-kDa FITC-dextran, at day 14 (fig. S6), again demonstrated fully perfused vascular beds for flow-conditioned microvessels. At day 14, only a small fraction of static-cultured vessels are perfusable (see Fig. 4F). Moreover, comparing day 14 to day 7, in all cases, vessel area is reduced (Fig. 4G). However, the average reduction in area [100 × (day 14 − day 7)/day7] is lower for flow-conditioned vessels (~15% for Δ*P* = 3 mmH_2_O and 12% for Δ*P* = 7 mmH_2_O, in contrast to a 20.5% decrease in static cultures). Permeability to 70-kDa dextran slightly increases in comparison to earlier time points (Fig. 4H, with the exception of the few static vessels that were perfused). Overall, the decrease observed in vascular coverage (compared to no flow) is significantly reduced in flow-conditioned microvessels over time, even allowing for culture of connected vessels past 3 to 4 weeks (fig. S7). Long-term culture of statically cultured vessels is not possible, as they are nonviable beyond 2 weeks.

### Velocity and shear stress distributions in placental-like microvessels depend on flow conditions

First, as a proxy for fluid velocity measurements, fluorescent beads were introduced into the microvessels at day 7 and tracked by time-lapse microscopy (fig. S8, A and B). Mean velocities were significantly increased for flow-conditioned vessels (fig. S8C), corresponding to an increased effective diameter (Fig. 2D).

**Fig. 5.**
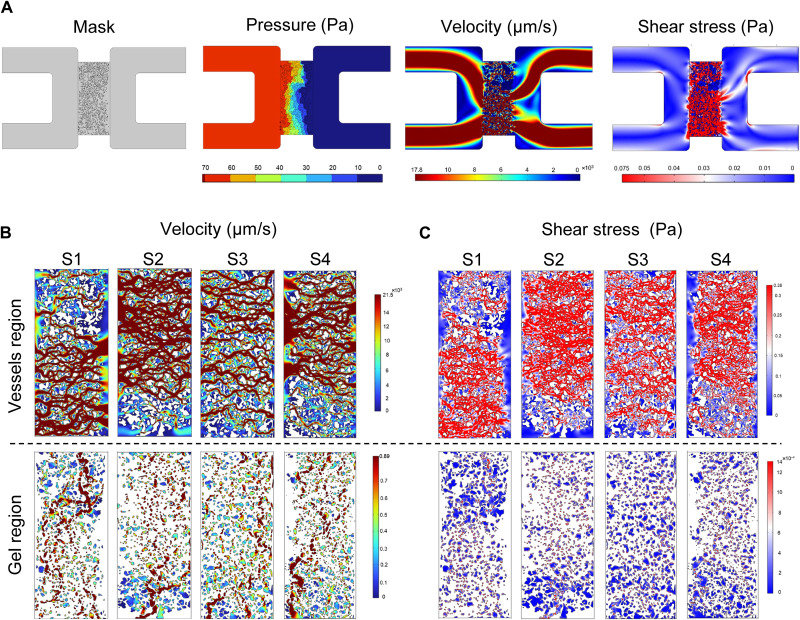
CFD predictions of fully perfused fetoplacental-like microvascular tissue. (**A**) Pipeline for computational predictions of velocities and shear stresses within microvessels embedded in a porous gel. Shown are four independent predictions from the 7-mmH_2_O condition for (**B**) velocity and (**C**) shear stress distributions within microvessels and gel regions.

Next, to accurately map velocity and shear stress distributions within our microvessels, large sections of 70-kDa FITC-dextran–perfused devices were imaged and converted into binary masks for import as vectors into CFD software. The computational modeling pipeline is outlined in Fig. 5A. Using COMSOL, both the vessels and extravascular regions were modeled as distinct, yet integrated, domains. Using the laminar flow physics module for flow-through porous media, microvessel networks are treated as open pores filled with cell culture media (density = 998.2 kg/m^3^; viscosity = 9.4 × 10^−4^ Pa·s). The EVM is treated as a porous domain with fibrin gel (density = 985 kg/m^3^, viscosity = 1 × 10^−2^ Pa·s) having a porosity of 0.3 and hydraulic permeability *k* = 1 × 10^−13^ m^2^ (*27*, *28*). To replicate the maximum pressure condition used in the experiment, a simulated pressure gradient of 70 Pa (7 mmH_2_O) was applied across the gel region. From these simulations, both velocity and shear stress distributions are predicted for the combined vessels and EVM domains (Fig. 5, B and C).

Estimated velocities and shear stresses for the complete tissue (vessels and gel combined) were lower overall than the vessel regions alone. Moreover, the gel regions demonstrate orders of magnitude lower values than the combined tissue (Table 1). CFD predicts heterogeneous flow patterns developed in these microtissues, which are contingent upon the transition from mostly interstitial at earlier time points to mostly luminal flows later on (Fig. 1E).

**Table 1.** Quantitative analysis of simulation outcomes. Surface averaging is applied to estimate the velocities and shear stresses (mean ± SD) within combined vessels + gel (tissue), vessels only, and gel only regions.

		Tissue	Vessel region	Gel region
Velocity (mm/s)	S1	11.22	15.39	0.89
	S2	17.78	21.54	0.90
	S3	9.64	13.83	0.73
	S4	11.54	15.04	0.89
Mean		12.55	16.45	0.85
±SD		3.11	3.00	0.07
		**Tissue**	**Vessel region**	**Gel region**
Shear stress (Pa)	S1	5.09 × 10^−2^	29.60 × 10^−2^	1.2 × 10^−3^
	S2	7.50 × 10^−2^	38.30 × 10^−2^	1.4 × 10^−3^
	S3	5.06 × 10^−2^	31.18 × 10^−2^	1.0 × 10^−3^
	S4	5.30 × 10^−2^	30.91 × 10^−2^	1.6 × 10^−3^
Mean		5.74 × 10^−2^	32.50 × 10^−2^	1.3 × 10^−3^
±SD		0.0102	0.0340	0.0002

### Flow conditioning alters biophysical properties of the placental-like microvascular tissue

**Fig. 6.**
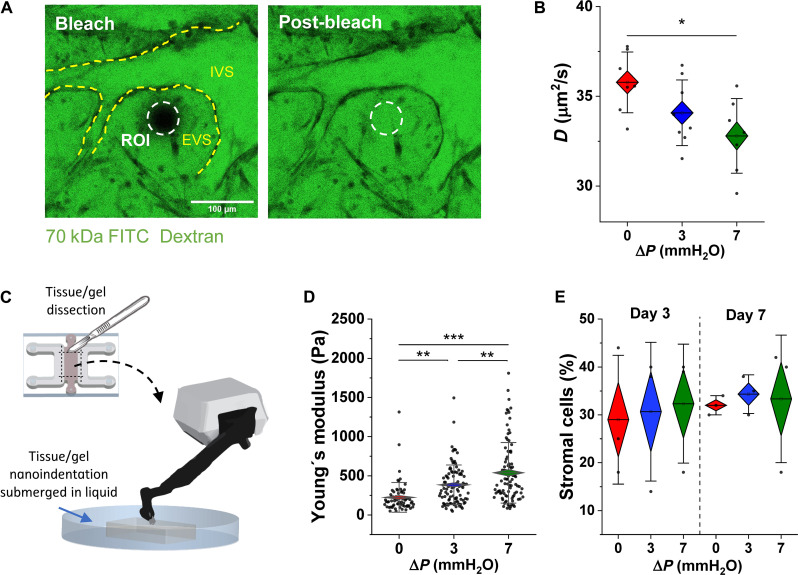
Interstitial flow alters extravascular remodeling and tissue-level stiffness. (**A**) FRAP measurements performed on extravascular regions. ROI is the bleached region of interest. EVS and IVS are extravascular and intravascular space, respectively. (**B**) Diffusivity measurements calculated from FRAP experiments for static and flow-conditioned microvessels at day 7. (**C**) Schematic representation of nanoindentation on microtissues. (**D**) Stiffness measurements performed across static and flow-conditioned samples. (**E**) Populations of stromal cells from microvessels corresponding to different culture times. Significance is measured by one-way ANOVA and indicated by *P < 0.05, **P < 0.01, and ***P < 0.001 for Tukey means comparison test.

The EVM supplies mechanical and chemical cues that contribute to vascular network formation and barrier integrity (*29*). We previously demonstrated significant changes in tissue stiffness and diffusivity in response to coculture with tissue-specific fibroblasts (*30*). Here, we assessed whether flow conditioning alone can drive these tissue-level changes. First, we examined diffusivity in extravascular regions from vessels cultured under static or flow conditions at day 7, as assessed by fluorescence recovery after photobleaching (FRAP) measurements (Fig. 6A). Flow conditioning, at high flow, results in significantly reduced diffusivity in extravascular regions (Fig. 6B).

Next, the impact of flow conditioning on microvessel tissue stiffness was assessed. Apparent Young’s moduli were assessed by nanoindentation at day 7 by exposing the tissue as shown in the schematic in Fig. 6C. Microvascular tissues cultured under flow are significantly stiffer than static tissues (Fig. 6D), with stiffness dependent on the magnitude of flow (7 mmH_2_O versus 3 mmH_2_O).

Because EVM is dynamic, constantly undergoing remodeling and able to activate cell proliferation and tissue morphogenesis (*31*), we assessed whether changes in tissue-level properties are associated with changes in the stromal cell population over time. To this aim, cells were extracted from the hydrogel matrix of static and flow-conditioned devices at 3 and 7 days after seeding to compare the cell composition over time. Cells were analyzed by flow cytometry (fig. S9) across days; however, no differences in the percentage of stromal cells were found (Fig. 6E).

### Flow promotes significant EVM protein deposition and remodeling

**Fig. 7.**
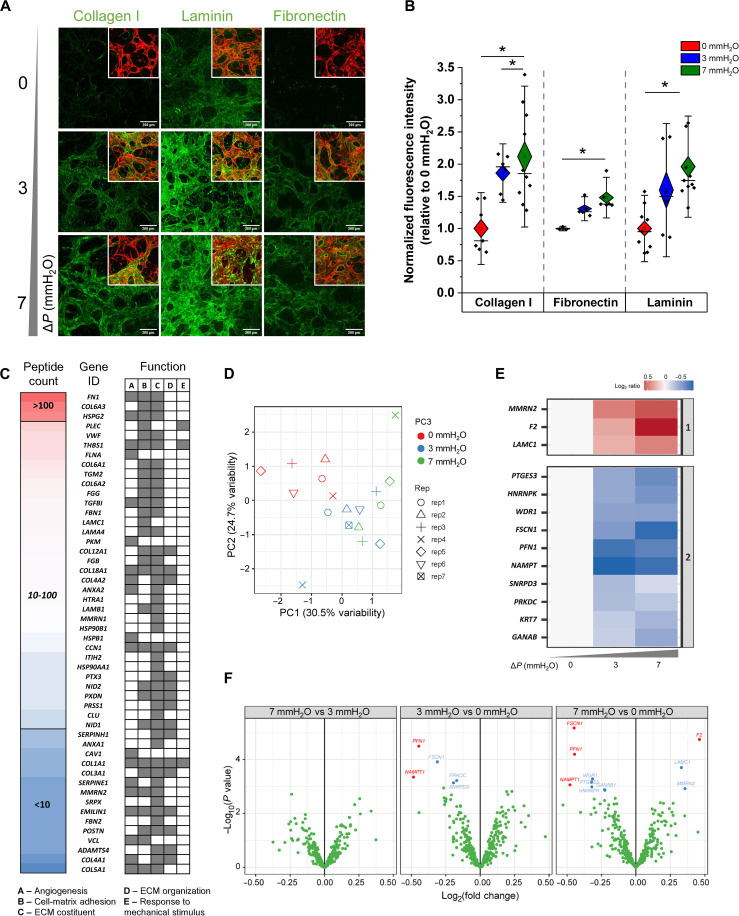
Flow induces changes in EVM protein deposition and composition. (**A**) Confocal images at day 7 of static and flow-conditioned microvessels (red labeling shown in the corner image insets) immunolabeled with collagen I, laminin, and fibronectin (green labeling). Scale bars, 200 μm. (**B**) Fluorescence intensity of collagen I, fibronectin, and laminin were measured and normalized to vessel area. Significance is measured by one-way ANOVA and indicated by *P < 0.05 for Tukey means comparison test. (**C**) EVM protein composition based on the total peptide count after 7 days of culture and ranked in a decreasing order of abundance (50 most abundant proteins). Proteins are categorized according to five ontology terms selected for their relevance to vascularization and matrix remodeling. (**D**) PCA performed on MS-based proteomics normalized data obtained from flow-conditioned samples for 7days. (**E**) *K*-means clustering and heatmap of the 13 hit and candidate proteins, showing differential expression across the static and flow conditions (shown as the log_2_ ratio of protein abundance in each 3- and 7-mmH_2_O sample relative to the average protein abundance in 0-mmH_2_O samples). (**F**) Volcano plots of the *P* values versus the log_2_ protein abundance differences between flow and static conditions. Red dots, hits: FDR < 0.05, FC > 30%. Blue dots, candidates: FDR < 0.05, no FC threshold. *P* values are calculated from moderated *t* test (limma).

Tissue-level stiffness increase corresponded with decreased diffusivity in extravascular regions of flow-conditioned placental vessels. Because there are many identified proteins in the developing placenta, we next examined the impact of flow on protein production. After 7 days in culture, microvessels were fixed and stained for collagen I, laminin, and fibronectin (Fig. 7A). In comparison to static conditions, all proteins assessed are more significantly expressed under flow conditions (Fig. 7B).

To assess the EVM more broadly, we performed mass spectrometry (MS) analysis to measure compositional changes of the matrix under static and flow conditions for vessels after 7 days in culture. Specifically, individual samples from each condition were subjected to tandem mass tag (TMT) labeling, followed by multiplexing, and ultimately measured in an MS-based experiment. As a result, we identified a total of 804 proteins, of which 333 were quantified (data S1), with many showing relevance to vascularization, EVM composition, and organization, as classified by gene ontology (Fig. 7C). For each protein, the fold change in expression between conditions was calculated, and ratios of protein expression were obtained (by dividing the flow-conditioned samples by the corresponding static counterparts). Principle components analysis showed that static and flow-conditioned sample replicates were clustered, consistently with their experimental condition (Fig. 7D). Thus, we identified 13 proteins with a false discovery rate (FDR) below 0.05, which were clustered into two different groups based on their expression similarities and behaviors under different conditions (Fig. 7E). No significant differences were observed between flow conditions. However, both the 3- and 7-mmH_2_O conditions exhibited notable variations in protein expression when compared to the static counterpart, as shown in the volcano plots analysis (Fig. 7F). In particular, we observed that flow-conditioned 7-mmH_2_O tissue exhibited increased expression of EVM components and known regulators of matrix stability such as prothrombin (*F2*, identified as a hit), laminin (*LAMC1*, candidate), and multimerin 2 (*MMRN2*, candidate). Conversely, we observed a significant decrease in the expression levels of the nicotinamide adenine dinucleotide biosynthetic enzyme nicotinamide phosphoribosyltransferase (*NAMPT*, hit), as well as proteins associated with actin dynamics and cytoskeleton organization, namely, profilin (*PFN1*, hit) and fascin (*FSCN1*, hit), in the presence of flow. These findings suggest that flow conditions can modulate the expression of key factors involved in EVM integrity and cellular cytoskeletal dynamics.

We lastly proceeded to assess the abundance of collagen 1, laminin, and fibronectin, which were found highly expressed in flow-conditioned cultures as demonstrated through immunostaining (Fig. 7, A and B). The MS assay identified the presence of fibronectin and various isoforms of collagen 1 (*COLA1* and *COLA2*), along with laminin (*LAMA4*, *LAMB1*, and the previously mentioned *LAMC1*), within the decellularized tissue matrices. Our data revealed a noticeable trend, indicating an increased presence of these proteins within the matrix when exposed to flow conditions (fig. S10), which closely mirrored the patterns observed in the immunostaining results.

## DISCUSSION

Limited access to human placentae, particularly at early stages of pregnancy, impedes the elucidation of mechanisms associated with fetoplacental vascular development. To circumvent this challenge, this study uses a 3D placental-like microvascular tissue on-chip. Building on our previous model (*25*), we generated an all-fetal derived vascular microtissue and incorporated a flow reservoir, allowing us to characterize vasculogenesis and extravascular remodeling processes in response to flow conditions. Incorporating placental fibroblasts, as opposed to coculture with lung fibroblasts as done previously (*25*), our model required parameter adjustment to achieve perfusable vessels. In this triculture, placental fibroblasts associate with microvessels, as do pericytes, as also shown in our previous model (*25*). Both studies have shown that stromal cells have a clear role in contributing to fetoplacental vascular growth and remodeling.

Besides what is known about the stroma, little is known about placental hemodynamics in early pregnancy, particularly in the villous vasculature. Ultrasound imaging has shown that blood flow velocity waveforms of the umbilical artery and its branches change with advancing gestation and correlate with the development of the placental villous trees and capillary networks (*32*). Despite the presence of a complete vascular network within the villi, it is believed that the chorionic circulation is not fully established until the end of the first trimester. This flow is progressively established in the third month of gestation, together with the perfusion of the maternal blood into the intervillous space (*33*). The precise mechanisms behind the formation and remodeling of the villous vascular network remain unknown. Nevertheless, we hypothesized that hemodynamic forces and associated signaling could play a role in directing vasculogenesis. Previous work has shown that IF promotes early vessel connectivity in 3D vasculature on-chip (*16*). Here, static, low, and high IF velocities ranging from ~0.1 to 1.2 μm/s (day 2 measurements) were applied via reservoirs (fig. S4). To the best of our knowledge, there are no reports on human fetoplacental interstitial fluid velocity; however, our measurements fall within the physiological range found in most soft tissues (from 0.1 to 4.0 μm/s) (*34*, *35*). For these placental-like vessels, IF applied at early time points is necessary to establish fully perfusable vascular networks, which transition to luminal flow by day 5 after seeding (Fig. 1E). Consistent with our observations, the flow conditions used in our experiments effectively induce vasculogenesis. This is evident from the shear stress experienced by the cells within the gel and the Peclet number (Pe), which were quantified using the equations described in (*13*). Our assessment revealed that the 3- and 7-mmH_2_O gradients generated shear stresses of 1.32 × 10^−5^ and 3.75 × 10^−5^ Pa, respectively. In addition, by considering the average interstitial fluid velocity (0.30 and 1.23 μm/s), the length of the central channel in the device (3 mm), and the diffusivity of a 70-kDa solute in the gel as measured by FRAP (*D* = 34.5 μm^2^/s), we determined that the interstitial fluid conditions corresponding to 3 and 7 mmH_2_O yielded Pe values of approximately 26 and 107, respectively. These Pe values exceed the reported threshold [Pe > 10 (*36*)] required to trigger a vasculogenic response.

Notably, there exists a subtle yet consistent distinction between the 3- and 7-mmH_2_O experimental conditions, in aspects such as vessels morphology, vascular permeability, and inflammatory signaling. Prior investigations have shown hyperactivation of endothelial cells and subsequent gel degradation at high pressure gradients (25 mmH_2_O) (*13*). Combined with our previous findings (*16*), our work supports the existence of a threshold to which vascular network are mechanoresponsive. Flow conditioning has a clear impact on vessel morphology, resulting in earlier connectivity, reduced branch density, and larger vessel diameters (Fig. 2). Endothelial barrier function significantly improved in high flow-conditioned vessels (Fig. 2E), compared to static cultures. Other studies conducted in perfused placental models have also demonstrated that shear stress (20 dyne/cm^2^ or 2 Pa) promotes vasodilatation (nitric oxide release) and decreases vascular resistance (*37*, *38*). Interstitial flow plays a critical role in the initial stages of vascular formation, facilitating processes such as sprout formation and vascular perfusion; however, it is low but present in the later development of microtissues, as predicted by CFD at day 7 (Fig. 5 and Table 1). Luminal flow becomes crucial in later stages, sustaining microvascular perfusability and longevity. In line with this, vascular abnormalities have been found in fetal growth restriction-affected placentae (*39*), which are known to be characterized by high vascular resistance and fetoplacental hypoperfusion. While speculative, our findings may implicate the need for flow early in fetal vessel development to prevent gestational complications.

Flow induces changes in vascular function and remodeling by promoting inflammatory and angiogenic responses (*40*, *41*); however, insight into flow-induced signaling in the placenta remains limited. Proinflammatory chemokines, including IL-8 and MCP1 are associated with angiogenesis, as evidenced by various studies (*42*, *43*). Sustained levels of these chemokines are produced by the placenta to stimulate the immune response against potential infections during pregnancy (*44*, *45*), but it remains unclear what role, if any, these chemokines play in the development of placental vasculature. Our findings indicate that IL-8 and MCP1 are strongly influenced by flow, aligning with previous reports of their regulation by shear stress (*46*–*48*). The human placenta has also been found to be a significant source of locally produced angiogenic factors, which include members of the VEGF family, fibroblast growth factor family, and angiopoietins, among others (*49*). We analyzed the levels of Ang2 in our fetoplacental-like vessels, which is highly expressed in early gestation and promotes vascular remodeling in the presence of VEGF (*50*, *51*). Our findings indicate increased Ang2 expression as a result of flow conditioning at early time points (Fig. 3C), which contrasts previous research showing a decrease in shear stress–dependent expression of Ang2 in experiments with flow-exposed HUVEC monolayers (*52*, *53*). It is worth noting that previous studies investigating Ang2 have primarily focused on laminar flow conditions [resulting in wall shear stress (WSS) >6 dyne/cm^2^ or 0.6 Pa], whereas other studies have demonstrated that Ang2 expression is up-regulated at lower shear stress levels (1 dyne/cm^2^) (*54*). Notably, the expression of Ang2 in fibroblasts has been previously found to elicit vascular growth, while concurrently maintaining a balance in the quiescent action exerted by Ang1, as produced by placental pericytes (*25*). These observations support our findings and highlight the need for further research to fully understand the complex relationship between shear stress and Ang2 expression. In particular, VEGF and its receptors are expressed in trophoblasts and villous fetal vessels at early developmental stages, suggesting their involvement in the initiation and progression of vasculogenesis (*55*). Here, we observed elevated VEGF levels at later stages (day 7) within static cultures, proposed as a compensatory mechanism to counteract the absence of flow. In line with this hypothesis, elevated VEGF expression was found in placentae from pregnancies with fetal growth restriction, suggesting that a decrease in fetal-maternal blood circulation during placentation enhances the expression of the angiogenic factor (*56*).

Considering that inflammation and angiogenesis are strongly linked to local oxygen concentrations, it was imperative to test microvessels in relevant O_2_ levels. Upon exposure to physiological oxygen conditions (8% O_2_), both vessel morphology and permeability closely paralleled those observed under standard atmospheric oxygen levels (in fig. S5). These findings provide compelling evidence for the remarkable ability of endothelial cells to adapt to varying oxygen gradients, with this adaptive response further enhanced by the preconditioning carried out before cell seeding in the experimental system. These results substantiate prior research findings (*57*), underscoring the critical importance of prolonged exposure to physiologic normoxia, as opposed to brief encounters with low-oxygen conditions, when striving to construct a model that faithfully replicates the in vivo microenvironment during physiological conditions.

Vessel stabilization is driven by hemodynamic forces through regression and pruning of branches exposed to low blood flow and by maintenance of vessel connections above a shear flow threshold (*58*). After 2 weeks of culture under intermittent flow, placental-like microvessels were perfusable and functional (maintained a relatively low permeability; Fig. 4 and fig. S6), consistent with observations in a brain 3D microvascular system (*23*). Flow conditioning exerted a significant influence on the attenuation of vascular constriction (Fig. 4G). In static conditions, there was a substantial decrease in area coverage, dropping from ∼74.5% to ∼59% from days 7 to 14. In contrast, under flow conditions, this reduction was less prominent, with values decreasing from ∼79% to 69.5% at 7 mmH_2_O and ∼67.4% at 3 mmH_2_O. Previous observations in HUVEC and lung cocultures have also shown that continuous flow (at a low WSS of <10 dyne/cm^2^ or 1 Pa) maintains a stable vascular diameter in formed vessels (*16*). At this late stage of culture, very few static-cultured vessels were perfusable (Fig. 4F), and those few vessels maintained a high barrier function (Fig. 4H, striped diamond box). We hypothesize that these few vessels comprise the least resistant pathway to fluid exchange and are exposed to shear stress upon media change. In the absence of flow, vessels were not viable beyond 2 weeks. Flow conditioning was essential in maintaining microvessel connectivity over 4 weeks in culture (fig. S7), highlighting the importance of implementing flow for long-term in vitro microvessel studies.

Particle tracking demonstrated a significant increase in the mean velocity in flow-conditioned vessels by day 7 (fig. S8). More accurate predictions were generated in COMSOL (Fig. 5). Heterogeneous flow distributions were apparent in the microvascular networks, which depend on the formation of open lumens in the media channels. Shear stress for flow-conditioned vessels was predicted to be 0.32 Pa (3.2 dyne/cm^2^), whereas shear stress within the EVM was 0.001 Pa (0.01 dyne/cm^2^). CFD results deviated from experimental bead measurements by approximately one order of magnitude. Note that velocities and shear stress predictions are obtained using 2D segmented projections and do not account for the exact spatial complexity of 3D vessels nor the wall effects or size-dependent effects of beads. It was reported previously that the presence of common additives in the media (*59*), tracer beads size, and their properties (*60*), such as deformability, contribute to elastic no-slip boundaries at the fluid interfaces and influence the velocities.

We postulated that differences in vascular morphogenesis and longevity, observed in the presence of flow, could be due to extravascular rearrangements and changes in the EVM, as previously reported (*61*). Recent findings showed that flow-conditioning reduces proteolytic activity of cathepsins prolonging in vitro microvessel stability (*62*). Flow-conditioned vessels, as opposed to the static ones, exhibited significantly reduced extravascular diffusivity and overall increased matrix/tissue rigidity (Fig. 6). Despite evidence showing that stromal cells can contribute to tissue stiffening (*30*), the flow-dependent increase in stiffness is not attributable to alterations in stromal cell population because they remain stable ( ~30% of the total cell population) over time (Fig. 6E). Examination of proteins in the EVM by immunostaining revealed that flow promotes increased deposition of collagen I, fibronectin, and laminin (Fig. 7, A and B). Several studies have reported the effect of shear stress in regulating matrix deposition and remodeling (*63*–*65*) but none in the context of placental tissue. Collagens, fibronectin, and laminins are abundant in the placental stroma and basement membrane, and their expression increases with advancing gestation to support the developing tissue structure and function (*66*). Low expression of these EVM proteins, observed in the absence of flow, could explain their increased destabilization over time. A recent study reported that endothelial cells plated on matrices derived from villous stromal fibroblasts where fetal growth restriction was clinically observed (reduced expression of collagen I and fibronectin) exhibited impaired proliferation and migration, suggesting that matrix composition is crucial for fetoplacental vascular development (*67*).

A more in-depth examination of the gel/tissue composition using MS revealed an abundance of EVM-related proteins deposited by the cells over the culture period (Fig. 7C). As expected, tissue protein enrichment is influenced by the presence of the flow, revealing changes in protein expression associated with EVM organization and vascular homeostasis. Consistent with the immunofluorescence findings, MS analysis revealed significant enrichment in laminin and a notable trend of increased abundance of collagen I and fibronectin in response to flow (fig. S10), indicating flow-induced adaptation and alterations in cell matrix adhesion, which ultimately resulted in matrix remodeling. In addition, multimerin 2, an EVM molecule that enhances vascular stability and regulates permeability by strengthening endothelial junctions (*68*, *69*), was found enriched in flow-conditioned tissues, suggesting a protective function in maintaining vessel function and longevity under mechanical shear stress. The substantial increase of prothrombin, which serves as a precursor to thrombin and facilitates fibrin formation (*70*), provides further evidence of flow-induced enhancements in EVM rigidity and subsequent matrix stabilization. Flow conditions also resulted in the depletion of NAMPT, an essential coenzyme that plays a critical role in energy production, DNA repair, and signaling pathways. The overexpression of NAMPT has been associated with inflammatory processes and the development of various human conditions, including acute lung injury, atherosclerosis, and cancer (*71*). While direct evidence of the specific effects of flow on NAMPT expression is lacking, our findings suggest that the protein is mechanosensitive, indicating a potential protective role in response to flow mechanical forces. Moreover, flow has an impact on actin dynamics, as indicated by the reduced levels of profilin and fascin in flow-conditioned tissue. On the basis of previous reports (*72*–*74*), we propose that the depletion of these actin-bundling proteins can alter the organization of the cytoskeleton and hinder cell migration. Consequently, once the perfusable vascularized tissue is formed, the absence or reduction of cellular movement plays a role in ensuring the functionality and longevity of the fetoplacental vascular barrier.

Although fetal and from the placenta (*75*), HUVECs are not chorionic endothelial cells. Other studies highlighted significant transcriptomic distinctions between placental macro- and microvasculature, as well as between placental and umbilical endothelium (*76*). Despite the use of HUVECs herein, our model incorporates placental-derived stromal cells in a 3D microenvironment, enabling the development of fetoplacental-like microvessels. Plasticity of endothelial cells is now well recognized (*77*–*79*), and recent evidence shows that 3D culture with organotypic stromal cells can modulate endothelial transcriptomics to closely match organ-specific phenotypes (*77*). Herein, placental-derived stromal cells contribute to the development of fetoplacental-like vessels, which are phenotypically different from our previously published triculture with nonspecific lung microvessels (*25*). Moreover, MS revealed proteomic profiles (data S1) closely resembling those obtained from placental decellularized explants (*80*). We also recognize the need for caution when extrapolating our findings to the early stages of placental development because cells used in this study were derived from term placentae. This model closely emulates the process of vasculogenesis, which is relevant during the first and second trimesters, but also at term, where vascular placental growth corresponds with fetal growth (*81*). It is also important to underscore the potential impact of donor variability on experimental outcomes, and the importance of placental-immunological factors, both of which were not explored herein.

To the best of our knowledge, this study is the first to examine the effects of flow on the formation of fetoplacental-like microvessels and modulation of their extravascular matrix. Although intermittent flow only partially reproduces the hemodynamic forces in vivo (*19*, *82*), the predicted shear stress generated in our system is sufficient for inducing perfusable, branched fetal-vascular networks that are stable for several weeks in culture. Overall, these fetoplacental-like microvessels demonstrate the importance of shear flow in placental vascular development. Although they lack the branched villous structure and critical epithelial layer (trophoblasts) that forms a barrier between the maternal blood and fetal capillaries, this model will be useful for future investigation of trophoblast-fetovascular cross-talk.

## MATERIALS AND METHODS

### Cell culture

All the cells used in this study were commercially sourced. HUVEC were purchased from Lonza (pooled donors) and cultured in endothelial media (VascuLife, Lifeline Cell Systems) on T75 or T150 flasks coated with rat tail collagen I (50 μg/ml; Roche). HUVEC were transduced to stably express cytoplasmic red fluorescent protein (RFP; LentiBrite RFP Control Lentiviral Biosensor, Millipore Sigma-Aldrich) and were used between passages 6 and 9. Primary human placental fibroblasts were purchased from American Type Culture Collection (ATCC; male donor), cultured in fibroblast media on uncoated T75 flasks (FibroLife, Lifeline Cell Systems) and used between passages 4 and 8. Unlabeled and green fluorescent protein–labeled primary human placental microvascular pericytes were acquired from Angio-Proteomie (male donor), cultured in pericyte growth medium according to the manufacturer’s protocols and used between passage 6 and 9. All cells were cultured under 21 or 8% oxygen conditions at 37°C and 5% CO_2_ in a standard incubator; dissociations were carried out using TrypLE Express (Gibco), and medium was completely refreshed every other day.

### Device fabrication

Devices were fabricated as previously described (*25*, *83*). Briefly, polydimethylsiloxane (PDMS) (SYLGARD 184 Silicone Elastomer Kit, Dow) was mixed at a 10:1 elastomer to cross-linker ratio according to the manufacturer’s protocols, degassed, and poured onto a mold. Following further degassing, PDMS was placed in a 60°C oven overnight, and single devices were cut, punched using 1- and 2-mm diameter biopsy punches for the gel and media ports, respectively, and air-plasma bonded (Harrick systems) to clean glass slides. Assembled devices were then incubated at 60°C overnight to restore the native hydrophobic state. All devices were sterilized under ultraviolet light for at least 30 min before cell seeding.

### Device seeding and microvessels formation under IF

A detailed methodology of the device seeding process can be found in our published book chapter (*83*). Briefly, fibrinogen derived from bovine plasma (Sigma-Aldrich) was reconstituted in phosphate-buffered saline (PBS) to a working concentration of 7 mg/ml before use. Thrombin (Sigma-Aldrich) stock solution [100 U/ml in 0.1% (w/v) bovine serum albumin solution] was diluted to a working solution (4 U/ml) in cold VascuLife medium. Endothelial cells and stromal cells were cultured until near confluence before detachment and resuspended in thrombin, separately, to concentrations of 24 million endothelial cells/ml and 2.4 million stromal cells/ml (a total combination of fibroblasts at 1.2 million/ml and pericytes at 1.2 million/ml). Cell suspensions were mixed 1:1 by volume and then combined with fibrinogen solution to produce a final concentration of 6 million endothelial cells/ml and 0.6 million stromal cells/ml, in a 10:1 ratio within fibrin (3.5 mg/ml). The cell-gel mixture was injected into the device central channel and allowed to polymerize for 15 to 20 min at 37°C in a humidified chamber. The cell-gel mixture is centrally contained by means of two partial walls that prevent the gel from perfusion into the adjacent channels. This pillar-free approach to gel containment facilitates stochastic vascular lumen formation at the gel-liquid interface. VascuLife medium was added to each media channel (150 μl total), and devices were initially cultured under static conditions. An intermittent IF across the hydrogel channel was generated 48 hours after seeding by adding 3D printed (Form3 printer, Formlabs) tailored media reservoirs (fig. S2; the design file can be provided upon request to the authors). Equal total volumes of media were differentially added to the reservoirs compartments to generate three hydrostatic pressure gradients (Δ*P*): 0, 3, and 7 mmH_2_O. The pressure gradients were reestablished with fresh media every day for 5 days following seeding. From day 8 onward, with the vessels fully perfusable, hydrostatic pressure gradients were restored every other day.

### Imaging and vessel morphology quantification

Three areas were selected along the middle axis of the central channel (vascular bed) of each device for morphology assessment, with the intention of mitigating any potential side effects or variations associated with pressure gradients between the left and right sides. All images were acquired on a Stellaris 8 confocal microscope using LAS X software (Leica). Confocal *z*-stacks were obtained for all time points and used to quantify the morphology of the microvascular networks cultured under different Δ*P*, as previously described (*25*). Briefly, a custom macro [ImageJ, National Institutes of Health (NIH)] was generated to process the images as follows: projection of maximum intensity of the RFP channel in the *z* direction, Gaussian filter smoothing, removal of outliers, and conversion to binary images. Particle analysis and 2D skeletonization were performed using built-in ImageJ plugins to determine branch density, network connectivity, and effective diameter (vessel area/total length).

### Permeability measurements

Microvessels cultured under different flow conditions were perfused at day 7 or day 14 with 70-kDa FITC-labeled dextran (Merck) by the application of a hydrostatic pressure drop across the central gel channel. Briefly, medium was removed and replaced by adding 40 μl of fluorescent perfusate solution (FITC, 0.1 mg/ml) into one channel. After perfusion into and through the microvessels, convective flow was stopped by applying an equal volume of dextran solution in the opposite channel. Following stabilization (~2 to 3 min), time-lapse confocal images were captured (3 × 5 min intervals), from which permeability measurements were made, as reported previously (*25*).

### Cytokine release

Supernatants were collected from three device reservoirs per Δ*P* condition of two or three independent experiments at days 3, 5, and 7 and were frozen until use. Concentration of VEGF, Ang2 and MCP1 were measured from individual samples following the manufacturer’s protocols of respective Quantikine ELISA Kits (R&D systems, DVE00, DANG20, and DCP00). Cytokine expression profiles of 2D cultures were assessed using a human angiogenesis antibody array (Abcam). Supernatants from lung fibroblasts, placental fibroblasts, and pericytes were collected 48 hours after seeding and processed according to the manufacturer’s instructions. Cytokine profiles on the array membranes were detected by chemiluminescence using the FUSION FX Spectra (Vilber, France). The relative (semiquantitative) expression of intensity was normalized to the positive controls.

### Fluid dynamics characterization by bead-tracking

Evaluation of fluid velocity of static and flow-conditioned vessels was assessed at day 7 through perfusion of 2.0-μm fluorescent beads (Fluorescent blue latex beads; Sigma-Aldrich) into the microvascular networks using reservoirs with applied pressure gradients of 5 mmH_2_O. Time-lapse images (1024 × 1024 pixels at a resolution of 0.64 μm per pixel with a 0.06-s frame rate) were acquired for 25 s using a fluorescent microscope (Thunder Imager–DMi8 microscope, Leica). Tracking of each individual bead (distance over time) was done using the particle tracking plugin, TrackMate (NIH ImageJ) (*84*). First, individual beads are detected on the basis of size through the “LoG detector” method with an “Estimated blob diameter” of 5 μm and an intensity threshold of 2 arbitrary units (AU). Then, the “simple LAP Tracker” method with “Linking max distance” of 15 μm, “Gap-closing max distance” of 15 μm, and “Gap-closing max frame gap” of two frames, is used to identify the same object over time. Upon application of a filter for “Track displacement” to easily remove nonmoving objects, the function “Analysis” is used to obtain the mean speeds of the beads.

### Computational fluid dynamics simulation

To estimate the range of fluid velocities and shear stresses generated by the application of pressure-induced flows within microvessels and surrounding gel domains, binary masks were obtained from confocal microscopy images and converted to drawing exchange format (dxf) in Adobe Illustrator 2021. These .dxf files were imported into COMSOL Multiphysics software (version 6.0). Using the laminar flow physics module with porous domain enabled physics setting, the spatial profile of luminal and interstitial fluid velocities and shear stresses were computed for 70-Pa applied pressure (a pressure drop, Δ*P*, of 7 mmH_2_O) between the inlet and outlet of four microfluidic chips containing placental microvessels. A computationally efficient physics-controlled extra fine mesh was applied to the model, resulting in an overall computational time of ~2 hours.

### Diffusivity measurements and initial fluid velocity tracking

Diffusivity was assessed in the extravascular space (adjacent but outside of vessels observable in images) for each flow condition using FRAP measurements on a Stellaris 8 confocal microscope (Leica). Devices were perfused with 70-kDa FITC labeled dextran and incubated at 37°C for 12 hours to allow total diffusion throughout the gel matrix (extravascular space). Small regions (30 μm Ø) within the matrix were bleached, and immediately after photobleaching, time-lapse images were recorded every 0.4 s to capture the fluorescence recovery. Analysis of diffusivity was performed using the MATLAB frap_analysis plugin (*85*), as previously described (*30*). Similarly, we determine the initial IF velocity (day 2 after seeding) using FRAP as done previously (*16*). Briefly, pressure gradients were generated across the gel and cell containing devices by adding FITC dextran to the corresponding reservoir media volumes. Following dextran saturation, a region of 30 μm Ø was bleached, and consecutive time-lapse images were collected to observe the fluorescence recovery following the direction of fluid flow. The IF velocity was estimated using the MATLAB frap_analysis plugin to track the movement of the centroid of the bleached region.

### Microtissue stiffness assessment

Mechanical resistance of fetal placental vascularized microtissues cultured under different flow conditions was measured on day 7 using a Chiaro Nanoindenter (Optics 11, Amsterdam, Netherlands), as previously described (*30*). Briefly, gels were cut and extracted from the devices, placed in a petri dish, and fully submerged in VascuLife media. Nanoindentation measurements were performed using a spherical probe tip with a radius of 25 μm and a stiffness of 0.027 N/m. After proper calibration of the probe, 12-μm-depth indentations were applied to the gels. The effective Young’s modulus values were derived from load-indentation curves by fitting to the standard hertz model and assuming a Poisson’s ratio of 0.5, using the manufacturer’s data analysis plug-in. An average of ~80 measurements were performed per condition, for two independent experiments.

### Flow cytometry

Characterization of stromal cell population was performed at days 3 and 7 after seeding by flow cytometry. To extract the cells from the devices, the gels were resected and digested in a solution of Accutase (Gibco) and Nattokinase (50 FU/ml; Japan Bioscience Ltd.) for 15 to 20 min at 37°C. An average of six gels was pooled for each time point. Single cells were then stained with the pericyte marker CD140b PerCP-Cy5.5 (BD Bioscience) for 2 hours at 4°C, washed with PBS, analyzed on a BD LSR II and later processed using FlowJo v.10.8.1 software. The stromal cell population was gated using CD140b^+^ and by exclusion of the RFP^+^ cell cluster (expressed only in HUVEC).

### Immunostaining

On day 7, devices were washed with PBS and fixed with 4% paraformaldehyde for 15 min. Samples were then permeabilized and blocked for nonspecific binding with 0.1% (v/v) Triton X-100, 5% (w/v) bovine serum albumin (BSA), and 1% (v/v) serum (same source of secondary antibody) in PBS overnight at 4°C on an orbital shaker. Primary antibodies were diluted in 0.5% (w/v) BSA PBS and added to samples overnight at 4°C on an orbital shaker. Primary antibodies used in this study are S100A4 (FSP-1; 1:100; Abcam), collagen I (1:200; Abcam), fibronectin (1:200; Abcam), and laminin (1:200; Abcam). The following day, samples were washed with 0.1% (v/v) Triton X-100 PBS and then incubated with the appropriate secondary antibody (1:200; Alexa Fluor 488 or 647; Invitrogen) and 4′,6-diamidino-2-phenylindole counterstain diluted in PBS overnight at 4°C on an orbital shaker. Samples were washed with PBS and stored at 4°C before imaging. To ensure effective staining and washing of unbound reagents, all wash and incubation steps were performed while applying a pressure gradient across the gel.

### Mass spectrometric characterization of matrix-derived proteins

Tryptic digestion of hydrogel/tissue samples for their for MS analysis was performed at day 7 after seeding as described by Sawicki *et al.* (*86*). Two experiments were conducted, with each experiment having ≥2 replicates per static- or flow-conditioned devices. Samples were first decellularized to remove cellular structures and protein contribution. Briefly, extracted gel matrices were washed with PBS and wash buffer (100 mM Na_2_HPO_4_, 2 mM MgCl_2_, and 2 mM EGTA) prior incubation to a lysis buffer (8 mM Na_2_HPO_4_ and 1% NP-40) for 90 min at 37°C. Following additional washes (300 mM KCl and 10 mM Na_2_HPO_4_), decellularized gels were degraded with collagenase (50 U/ml in Hanks’ balanced salt solution; Gibco) for 1 hour at 37°C to release all retained proteins. Gels were then dissolved by vigorous pipetting and stored at −80°C before lyophilization (Labconco FreeZone 2.5). Lyophilized samples were reconstituted in 25 mM NH₄HCO₃, reduced with dithiothreitol and cysteine residues were alkylated by the addition of iodoacetamide (*86*). Proteins were digested over night by the addition of trypsin (Promega), and samples were acidified by using formic acid according to (*86*). Digested samples were applied to spin column concentrators (Corning) with a 10-kDa cutoff to remove trypsin and collagenase. Mixed peptides were subjected to a reverse phase clean-up step (OASIS HLB 96-well μElution Plate, Waters). Peptides were dried and reconstituted in 10 μl of 400 mM Hepes/NaOH (pH 8.5) and reacted for 1 hour at room temperature with 80 μg of TMT18plex (Thermo Scientific) dissolved in 4 μl of acetonitrile. Peptides were subjected to a reverse-phase clean-up step before their analysis by liquid chromatography–tandem MS (LC-MS/MS) on an Orbitrap Fusion Lumos mass spectrometer (Thermo Scientific).

To this end, peptides were separated using an Ultimate 3000 nano RSLC system (Dionex) equipped with a trapping cartridge (Precolumn C18 PepMap100, 5 mm, 300 μm inner diameter, 5 μm, and 100 Å) and an analytical column (Acclaim PepMap 100. 75 cm by 50 cm C18, 3 mm, 100 Å) connected to a nanospray-Flex ion source. The peptides were loaded onto the trap column at 30 μl/min using solvent A (0.1% formic acid) and eluted using a gradient from 2 to 80% solvent B (0.1% formic acid in acetonitrile) over 2 hours at 0.3 μl per min (all solvents were of LC-MS grade). The Orbitrap Fusion Lumos was operated in positive ion mode with a spray voltage of 2.2 kV and capillary temperature of 275°C. Full scan MS spectra with a mass range of 375 to 1.500 mass/charge ratio (*m*/*z*) were acquired in profile mode using a resolution of 120,000 with a maximum injection time of 50 ms, AGC operated in standard mode, and a RF lens setting of 30%. Fragmentation was triggered for 3-s cycle time for peptide like features with charge states of 2 to 7 on the MS scan (data-dependent acquisition). Precursors were isolated using the quadrupole with a window of 0.7 *m*/*z* and fragmented with a normalized collision energy of 34%. Fragment mass spectra were acquired in profile mode and a resolution of 30,000. Maximum injection time was set to 94 ms, and AGC target was set to custom. The dynamic exclusion was set to 60 s.

Acquired data were analyzed using FragPipe (*87*) and a UniProt *Homo sapiens* database (UP000005640, ID9606 with 20594 entries, 26 October 2022) including common contaminants. The following modifications were considered: carbamidomethyl (C, fixed), TMT18plex (K, fixed), acetyl (N-terminal, variable), oxidation (M, variable), and TMT18plex (N-terminal, variable). The mass error tolerance for full-scan MS spectra was set to 10 parts per million and for MS/MS spectra to 0.02 Da. A maximum of two missed cleavages were allowed. A minimum of two unique peptides with a peptide length of at least seven amino acids and a false discovery rate below 0.01 were required on the peptide and protein level (*88*).

### MS data processing

The raw output files of FragPipe [protein.tsv – files (*87*)] were processed using the R programming language (ISBN 3-900051-07-0). Contaminants were filtered out, and only proteins that were quantified with at least two unique peptides were considered for the analysis. A total of 333 proteins passed the quality control filters. Log_2_-transformed raw TMT reporter ion intensities were first cleaned for batch effects using the “removeBatchEffects” function of the limma package (*89*) and further normalized using the vsn package [variance stabilization normalization (*90*)]. Proteins were tested for differential expression using the limma package. The replicate information was added as a factor in the design matrix given as an argument to the “lmFit” function of limma. A protein was annotated as a hit with an FDR smaller than 5% and a fold change of at least 30% and as a candidate with an FDR below 5% with no fold change threshold. Hit and candidate proteins were clustered into two clusters (method *k*-means) based on the Euclidean distance between normalized TMT intensities divided by the 0-mmH_2_O data point.

### Statistics

Statistical significance was analyzed using OriginPro v.9.85 performing one-way analysis of variance (ANOVA) followed by the post hoc Tukey’s test for multiple comparison. Differences were considered statistically significant with *P* < 0.05. Data shown here are from at least two independent experiments with *n* ≥ 3 devices, with at least three measurements per device, unless otherwise specified.
