# Supplementary material for: Flow in fetoplacental-like microvessels in vitro enhances perfusion, barrier function, and matrix stability
Source: Sci Adv. 2023 Dec 22;9(51):eadj8540. doi: 10.1126/sciadv.adj8540 (PMC10745711; doi:10.1126/sciadv.adj8540)
Supplement: Supplementary file 1 — Figs. S1 to S10 Legend for data S1 [file sciadv.adj8540_sm.pdf]

Science Advances
AAAS

Supplementary Materials for

Flow in fetoplacental-like microvessels in vitro enhances perfusion, barrier function, and matrix stability

Marta Cherubini *et al.*

Corresponding author: Kristina Haase, kristina.haase@embl.es

Sci. Adv. **9**, eadj8540 (2023)
DOI: 10.1126/sciadv.adj8540

The PDF file includes:

Figs. S1 to S10
Legend for data S1

Other Supplementary Material for this manuscript includes the following:

Data S1

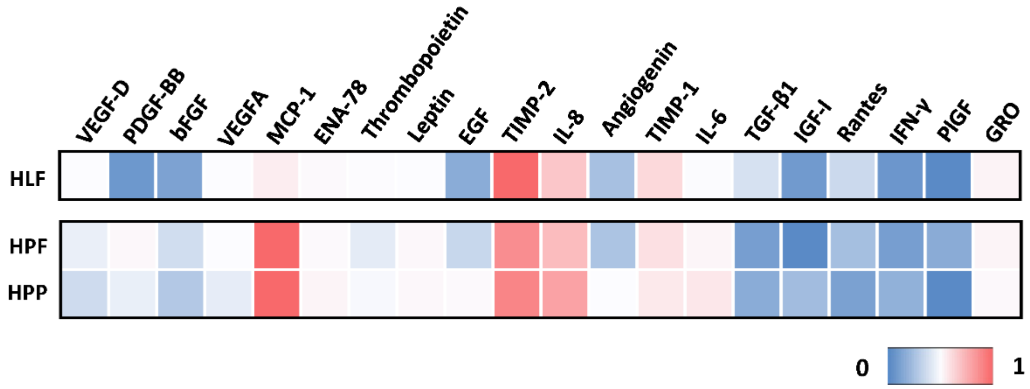

Figure S1. Cytokine expression of stromal cells in 2D culture. An array of cytokines is shown by relative expression levels for human lung fibroblasts (HLF), human placental fibroblasts (HPF), and human placental pericytes (HPP).

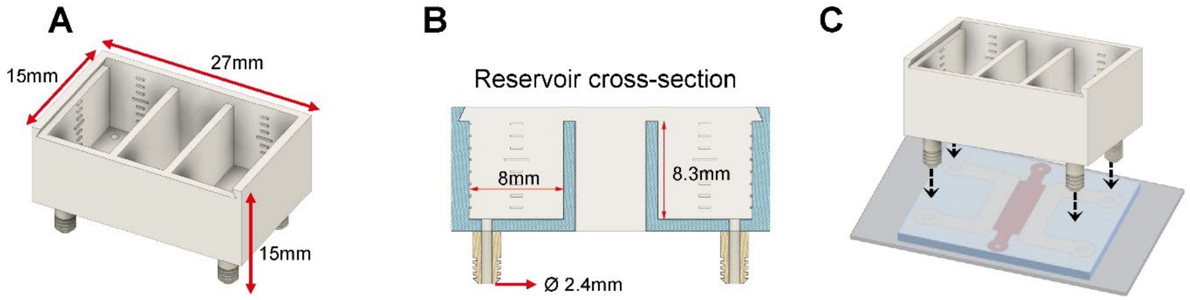

Figure S2. Reservoir design and application. A) 3D design of the media reservoir equipped with two media compartments and four interconnected feet. **B)** Frontal cross-sectional view of the reservoir showing the media containers and the interconnected feet with holes. **C)** The reservoir feet are securely inserted into the corresponding holes located on the side media channels of the device.

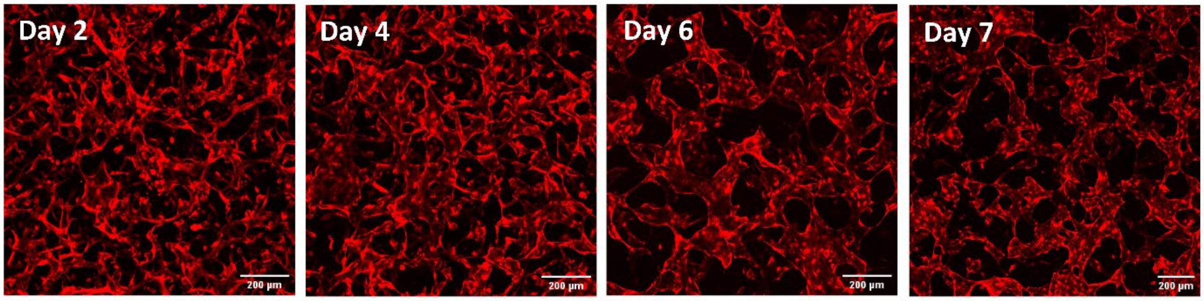

Figure S3. Fluorescent images demonstrating microvessel development in the absence of media reservoirs. Vessel connect and form without flow and reduced media volume; however, the connectivity is low as seen by day 6 and 7 and they are not perfusable. HUVEC are shown in red (cytoplasmic label).

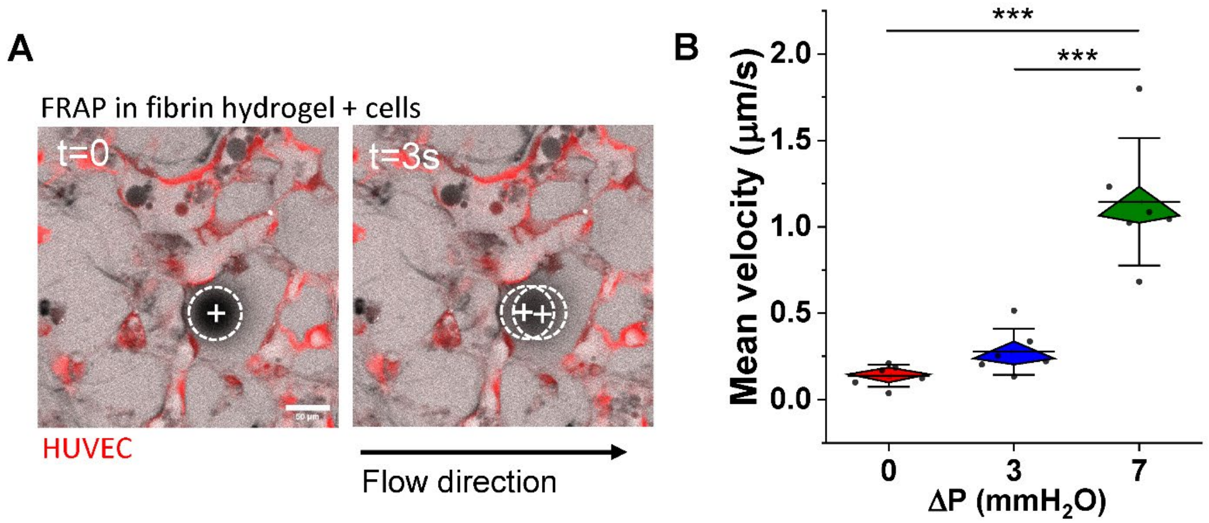

Figure S4. Assessment of interstitial flow velocity at day 2. A) FRAP measurements were performed in fibrin hydrogels with cells (tri-culture) to measure **B**) mean interstitial velocity for different fluid volume change (pressure difference across the gel). At this stage, vessels are not yet formed. Scale bar, 50μm.

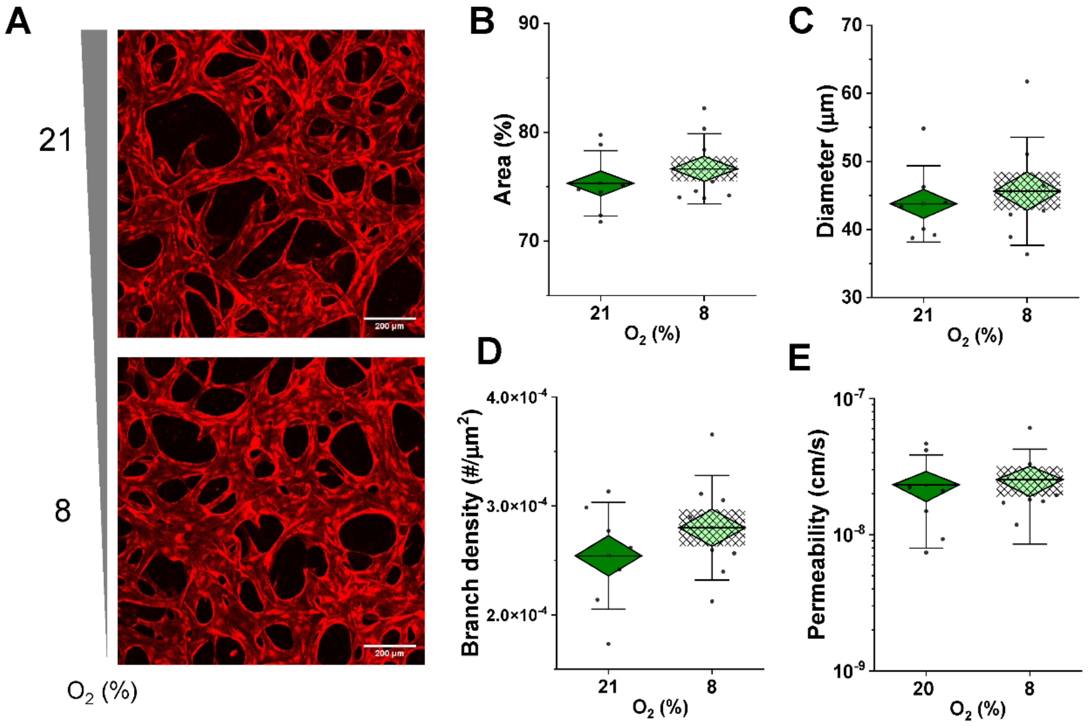

Figure S5. Fetoplacental-like vessels show similar morphology and permeability under physiological and atmospheric oxygen conditions. A) Confocal images show no differences in **B**) area, **C**) vessel diameter and **D**) branch density between 21 and 8% oxygen and flow-conditioned (7mmH_2O) vessels at day 7 in culture. **E**) Physiological low oxygen environment does not impact endothelial barrier function. Scale bars, 200µm.

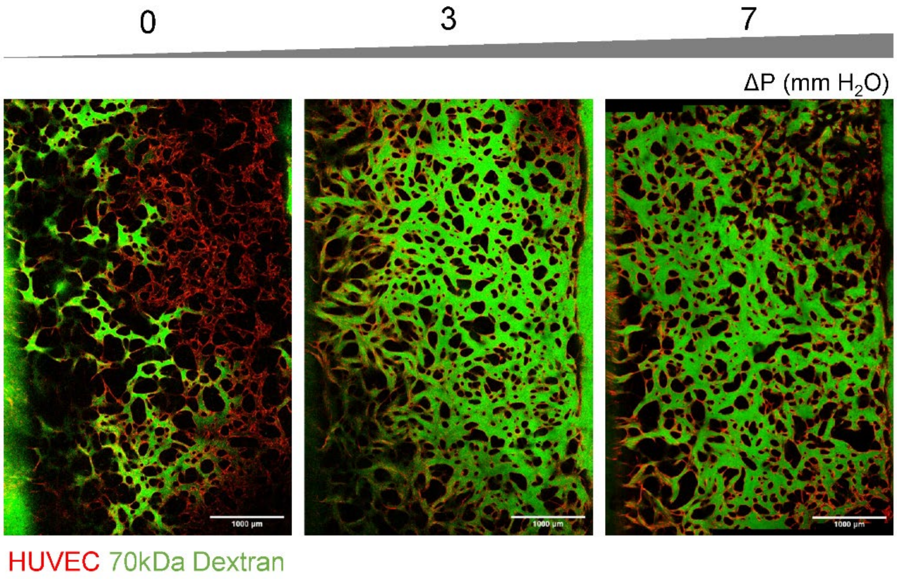

Figure S6. Long-term culture with flow maintains perfusion capacity of microvessels. Shown are overview images of example microvascular beds cultured under static and flow conditions demonstrating the complete perfusion for flow-conditioned vessels and only partial perfusion for static cultured placental vessels at day 14. Scale bars, 1000μm.

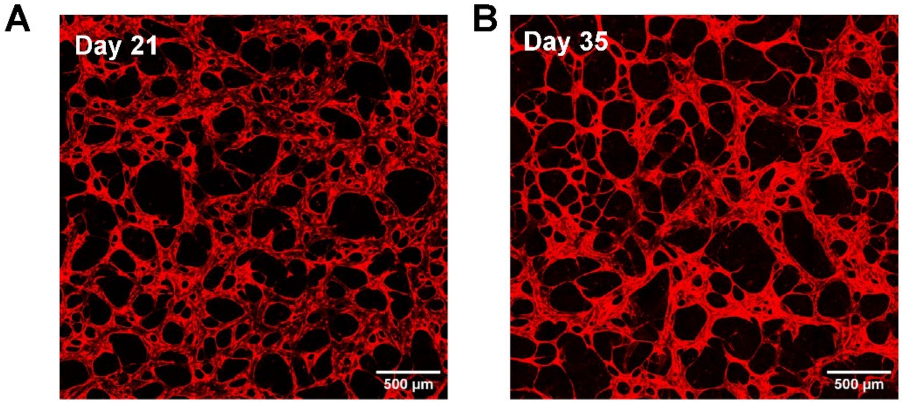

Figure S7. Intermittent flow-conditioning promotes vessels longevity. Flow-conditioning enables long-term stability of vessels to **A**) 3 weeks and **B**) past 4 weeks in culture. Beyond 4 weeks vessels remain connected but begin to narrow in diameter. HUVEC are shown in red (cytoplasmic label).

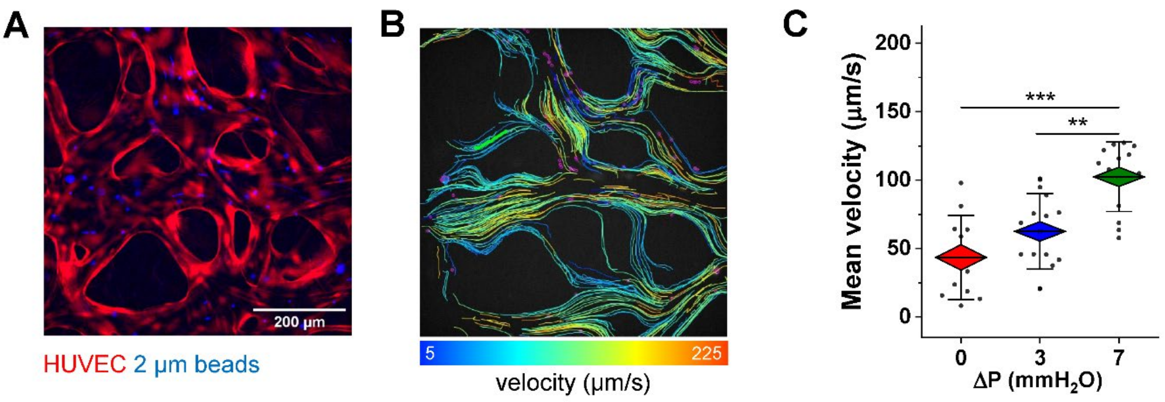

Figure S8. Characterization of luminal flow reveals heterogeneous patterns. A) Representative image of fluorescent tracer beads in lumen in day 7 vessels cultured under flow conditions (7 mmH_2O). **B**) Example of tracked paths from beads in A. **C**) Quantification of mean bead velocities from measurements in static and flow-conditioned microvessels subjected to a ΔP of 5 mmH_2O. Significance is measured by One-way ANOVA and indicated by $^{**}<0.01$, and $^{***} < 0.001$ for Tukey means comparison test.

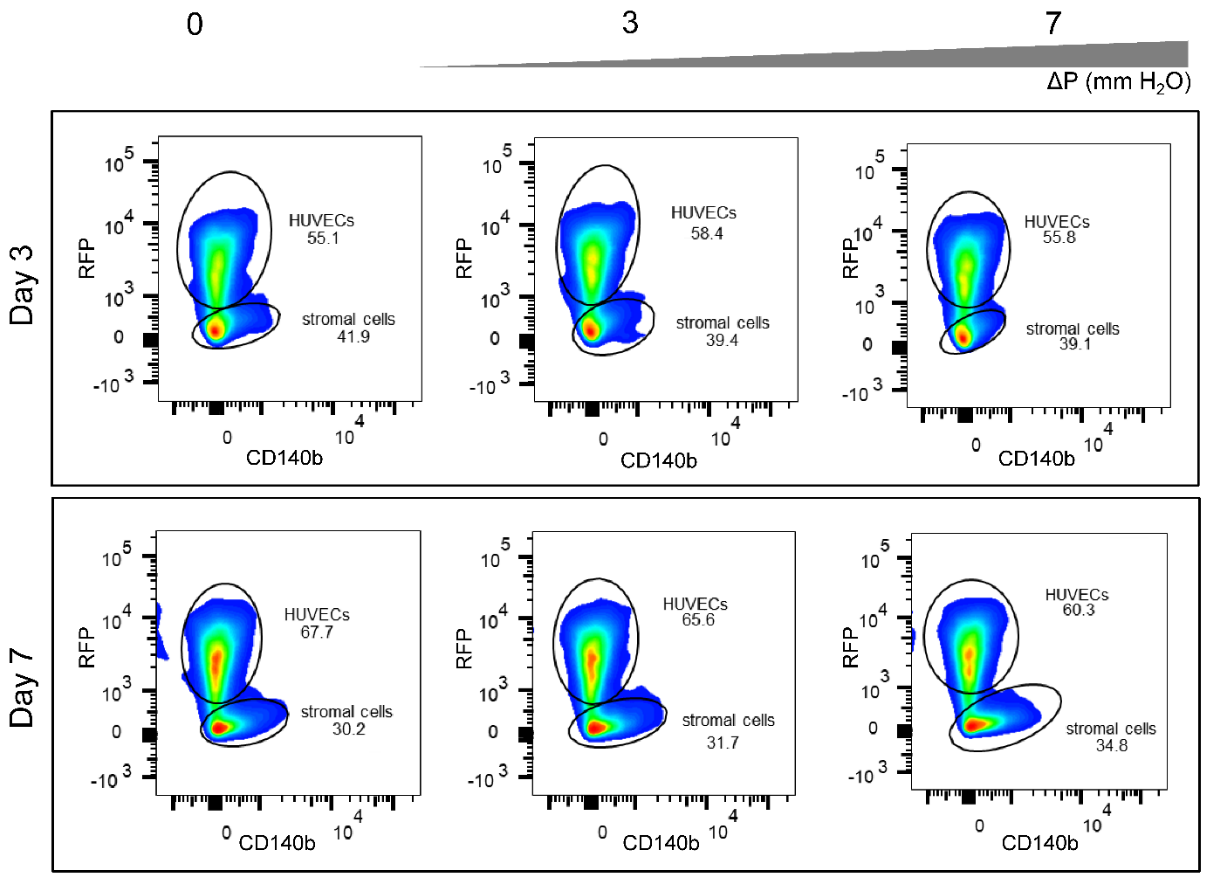

Figure S9. Flow cytometry of endothelial and stromal populations over time. Density plots are shown for static and flow-conditioned vessels at days 3 and 7. Stromal cell population was gated based on RFP- (expressed only in HUVEC) and CD140b (pericyte marker) expression. This figure represents one experiment, however 4 independent experiments were performed.

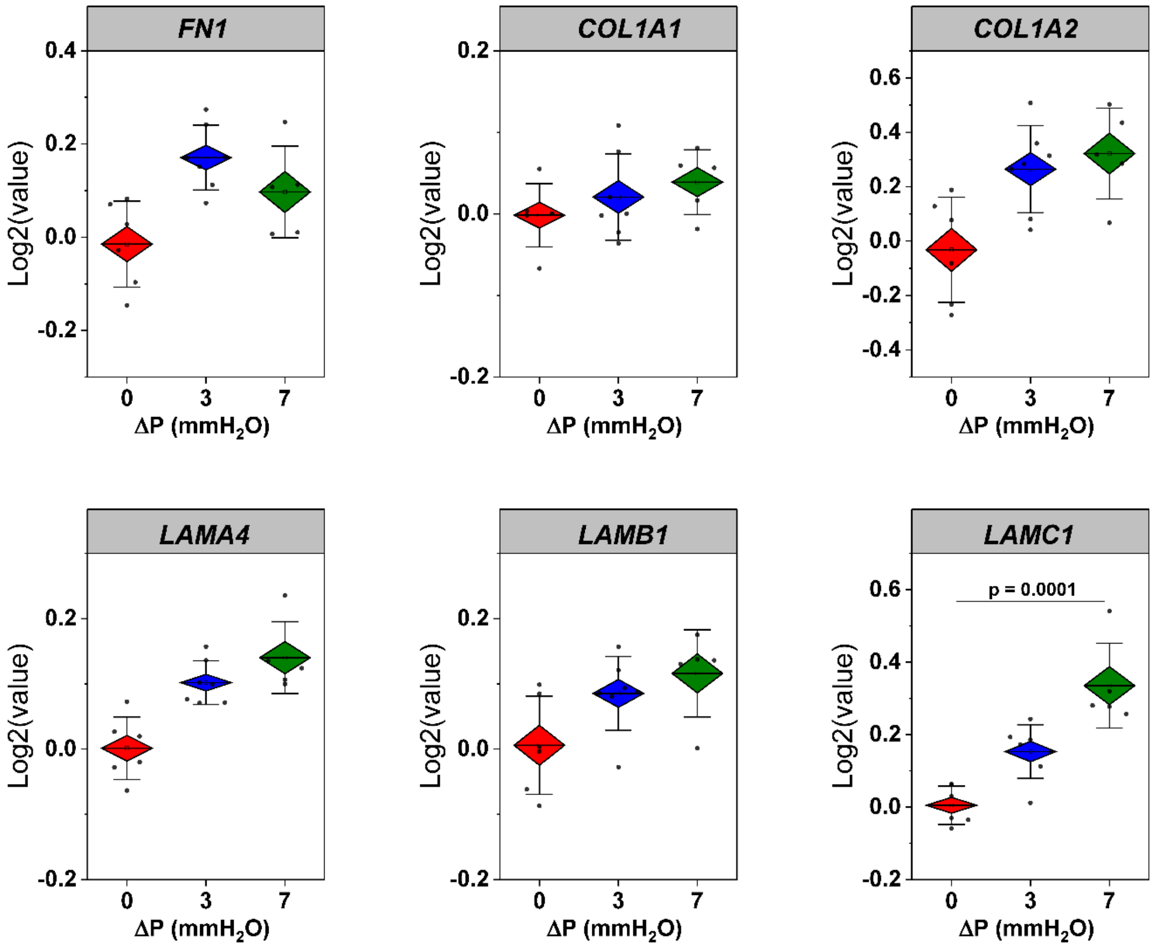

Figure S10. Protein abundance ratios of fibronectin, and various isoforms of collagen I, and laminin as identified through mass spectrometry analysis. The results reveal an increasing trend in protein abundance within tissue matrices cultured under flow conditions when compared to static counterparts.

Data S1. Comparative mass spectrometry data. List of identified protein and relative Limma analysis results.
